# Linear Ablation Using a Contact Force-Sensing Catheter in Ablation for Persistent Atrial Fibrillation: A Prospective Randomized Trial

**DOI:** 10.3390/jcm13237310

**Published:** 2024-12-01

**Authors:** Dae-In Lee, Kwang-No Lee, Seung-Young Roh, Yun Gi Kim, Jaemin Shim, Jong-Il Choi, Young-Hoon Kim

**Affiliations:** 1 Division of Cardiology, Department of Internal Medicine, Korea University Medical Center, Korea University College of Medicine, Seoul 02841, Republic of Korea; acttopia@gmail.com (D.-I.L.);; 2 Division of Cardiology, Department of Internal Medicine, Ajou University Hospital, Suwon 16499, Republic of Korea; knlee81@ajou.ac.kr

**Keywords:** atrial fibrillation, catheter ablation, contact force, linear ablation, cardiac magnetic resonance imaging

## Abstract

**Background/Objectives**: Pulmonary vein isolation (PVI) using radiofrequency catheter ablation with contact force (CF)-sensing technology has improved long-term outcomes in patients with atrial fibrillation. This prospective randomized study aimed to assess the efficacy and safety of CF-sensing technology for additional left atrial (LA) linear ablation of persistent AF (PerAF). **Methods**: After PVI, anteromitral (AM) line and roof line ablation were performed using a CF-sensing catheter. Patients were randomly assigned to either the CF-sensing (CFS) group or the CF-blind control (Blind) group. The primary endpoint was atrial arrhythmia recurrence. LA late gadolinium enhancement (LA-LGE) MRI was conducted at baseline and 1-year follow-up for long-term lesion evaluation. **Results**: A total of 62 patients with drug-refractory PerAF were enrolled (mean age: 58 ± 10 years; 77% male). The success rates of AM and roof line block were 97% and 100% in the CFS group (n = 33) and 93% and 90% in the Blind group (n = 29). The time to achieve block was reduced in the CFS group (AM: 36 ± 22 vs. 48 ± 28 min, *p* = 0.068; roof: 19 ± 14 vs. 27 ± 15 min, *p* = 0.031). The maximum CF for safety endpoints was significantly lower in the CFS group (AM: 42 vs. 69 g, *p* < 0.001; roof: 33 vs. 49 g, *p* = 0.003). Full linear LA-LGE formation on 1-year MRI did not differ significantly between the groups (AM: 17 vs. 36%; roof; 29 vs. 24%, both *p* = NS). Kaplan–Meier estimates of AF/AT-free survival after ablation procedures were 63.6% in the CFS group and 58.6% in the Blind group (log-rank *p* = 0.837). **Conclusions**: In patients with PerAF, additional LA linear ablation following PVI using CF-sensing technology improved procedural safety and reduced the time needed to achieve conduction block. However, it did not significantly influence clinical outcomes or the formation of permanent full linear lesions.

## 1. Introduction

Atrial fibrillation (AF) is the most common arrhythmia, leading to stroke, heart failure and increased mortality [1]. Based on data from the Framingham Heart Study, the prevalence of AF has increased 3-fold over the last 50 years [2]. Catheter ablation is currently regarded as an effective treatment option for symptomatic atrial fibrillation (AF) [1]. Pulmonary vein isolation (PVI) is the cornerstone of the procedure, but arrhythmia recurrence rates after PVI are still as high as before [3,4]. Additional strategies have been proposed to improve outcomes, but none showed consistently better results. So these additional strategies still vary according to the decision of the operator [5]. Left atrial (LA) linear ablation for electrical block is a treatment that was invented to prevent macroreentrant atrial tachyarrhythmia, which can occur after the procedure [6]. It comes from the Maze operation, a surgical strategy for AF, which is often used in the procedure for persistent atrial fibrillation (PerAF) [7]. Multiple studies have assessed the efficacy of linear ablation, but this procedure in addition to PVI did not show a difference in prognosis [6]. One of the reasons that LA linear ablation does not produce the ideal effect is due to technical difficulties. An electrical gap from incomplete block is common and an important cause of another reentrant atrial tachycardia [8,9].

Contact force (CF) monitoring, which enables the operator to accurately assess the pressure on the cardiac wall at the tip of the catheter, has become widely adopted in arrhythmia ablation procedures. This technology has demonstrated the advantage of significantly reducing both procedure time and the incidence of early reconnections during PVI [10,11]. Several studies have established a correlation between lower CF values and pulmonary vein (PV) reconnections, with CF-guided ablation improving the long-term durability of PVI [12]. CF-guided ablation consequently reduced AF recurrence rates after the procedure.

Although the anatomical targets for LA linear ablation are structurally less complex than the pulmonary veins, the ablation line is generally longer. Furthermore, ablation targets such as the anterior wall, the peri-mitral isthmus and the roof of the left atrium are thicker than the PV ostium or the posterior wall, potentially making transmural lesion creation more challenging. The relationship between CF values and the successful formation of linear blocks during LA ablation has not been definitively established. Importantly, previous investigations into the clinical efficacy of linear ablation were conducted before the widespread adoption of CF-sensing technology, and thus may not account for its impact on outcomes. In this randomized study, we aim to evaluate both the efficacy and safety of linear ablation utilizing CF-sensing catheters.

## 2. Materials and Methods

The left atrial LInear Block using Contact force-sensing catheter in Ablation for Atrial Fibrillation (LIBCAAF) study is a prospective, randomized, controlled, single-center study designed to evaluate the safety and effectiveness of CF-guided linear ablation in the treatment of symptomatic PerAF. Patients were enrolled in this trial after providing written informed consent. The process of obtaining informed consent adhered to the latest version of the Declaration of Helsinki, ISO 14155, and all applicable regulations. The trial protocol was approved by the ethics committee of the Korea University Anam Hospital Institutional Review Board. This study has been registered with ClinicalTrials.gov (NCT03091972).

### 2.1. Study Population

**Figure 1:**
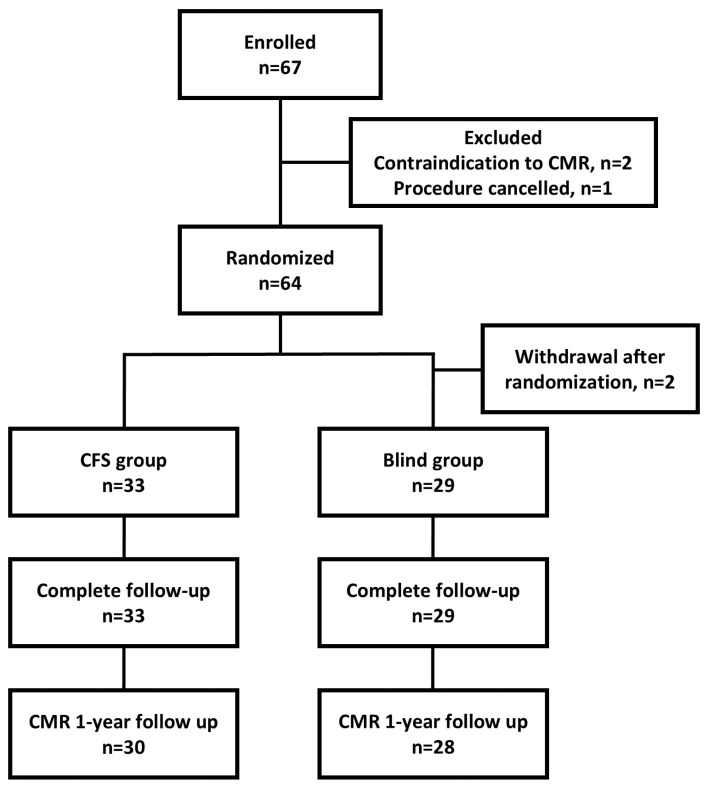
Study flowchart. CFS = contact force sensing; CMR = cardiac magnetic resonance.

All patients indicated for AF ablation were screened for eligibility. Inclusion criteria were as follows: (1) patients with drug-refractory PerAF defined as AF episodes lasting longer than 7 days and (2) patients undergoing a first-time ablation procedure for AF. Exclusion criteria were as follows: (1) prior LA ablation (either surgical or catheter), (2) left atrial anteroposterior diameter >60 mm, (3) left ventricular ejection fraction <35%, (4) the presence of moderate to severe valvular disease and (5) contraindication to cardiac magnetic resonance (CMR). After enrolment, participants were randomly assigned to either the contact force-sensing (CFS) group or the contact force-blind (Blind) group (Figure 1). We used web-based computer algorithms with random number generators to produce random values, which were then used to assign participants.

### 2.2. Catheter Ablation Procedure

PVI was performed during sinus rhythm. If AF persisted, rhythm was restored by internal or external cardioversion prior to PVI. Voltage mapping was performed in all patients before PV isolation. If AF recurred immediately after cardioversion, PVI was conducted in the AF state. The PV isolation procedure included the antrum of all four pulmonary veins. CF monitoring was utilized in both groups, and during PV isolation, the operator had access to all functions associated with CF monitoring. After confirming isolation of all the PVs with a Pentaray^TM^ catheter, linear ablation was attempted.

The linear ablation was performed in the following sequence: (1) roof line, (2) anteromitral (AM) line and (3) cavotricuspid isthmus (CTI) line. In the CFS group, all contact force data were visible to the operator with point-by-point time and force recorded via the Carto Visitag^TM^ module. In the Blind group, contact force was recorded but remained hidden from the operator, and ablation points were determined manually. Operators were unable to access CF-related data during linear ablation procedures on patients in the Blind group. The ablation energy for all lines was set at 30 W. Time spent on each ablation line and total fluoroscopic time were measured. Electrical block of the roof line was verified by placing the Pentaray^TM^ catheter on the posterior wall of the left atrium and assessing for the electrical signal reversal. This was reconfirmed by positioning the ablation catheter in the LA appendage and pacing to evaluate the conduction sequence across the posterior wall. Failure to achieve a complete block after one hour of roof line ablation was defined as a block failure. The maximum procedure time per person was set to eight hours. Based on this, time limits for procedural failure for each line were established in the procedure protocol. The AM line ablation was performed from the anterior aspect of the right superior PV to the anterior left side of the mitral valve. Successful block was confirmed when placement of the Pentaray^TM^ catheter in the LA appendage revealed a delay of more than 100 ms from the baseline signal.

Failure to achieve block within two hours of AM line ablation was classified as a block failure. CTI ablation was performed by placing a duodecapolar catheter in the coronary sinus (CS) and low right atrium (RA), with pacing from the CS ostium. A reversal of the lateral RA activation sequence was considered indicative of a block. Failure to achieve block within one hour was considered to indicate an unsuccessful procedure. In all patients, voltage mapping was conducted after the prescribed protocol was completed.

### 2.3. Follow-Up

Patients were followed for 12 months after the index procedure, with office visits scheduled at 7 days and at 3, 6 and 12 months post-ablation. At each visit, an ECG was performed for all patients, and 24 h ambulatory Holter monitoring was conducted at 6 months and at the end of the study. Atrial arrhythmia recurrence was defined as sustained atrial arrhythmia observed on a 12-lead ECG at any time or as lasting more than 30 s on Holter monitoring. Atrial arrhythmia recurrence was assessed by an independent clinician. Additional tests were performed if the patient reported symptoms or at the clinician’s discretion. All class I and III antiarrhythmic drugs (AADs) were discontinued within three months.

### 2.4. Cardiac Magnetic Resonance

Twelve months after the index procedure, CMR was performed to measure the late gadolinium enhancement (LGE) of the left atrium. The LA-LGE CMR protocol and LGE quantification method were detailed in our previous study [13,14]. CMR was performed on a 3-T MR system (ACHIEVA; Philips Medical Systems, The Netherlands) with a 32-element cardiac coil. Pulmonary vein (PV) MR angiography used a contrast-enhanced timing robust sequence after administering 0.2 mmol/kg gadolinium-based contrast (DOTAREM; Guerbet, France). High-resolution LGE-CMR images were acquired ~20 min post-contrast using a 3D inversion recovery, ECG-gated, respiration-navigated sequence. Typical acquisition parameters included a voxel size of 1.5 mm^3^, TR/TE 4.7/1.4 ms, TI 230–270 ms, a flip angle of 25°, and sensitivity encoding with R = 2. All images were transferred to a workstation for quantitative analysis. LGE measurements were performed by two physicians who were blinded to the patient groups, with discrepancies adjudicated by a committee. The roof line was divided into three sections, R M L, to evaluate the distribution of LGE. The AM line was divided into four sections (R, RM, LM, L). Permanent lesions were considered to have formed when LGE covered 90% of the lines in each section, and complete line formation was confirmed when lesions were present in all three roof sections and all four AM line sections. Ablation scar lines in CMR are three-dimensional lesion areas rather than strictly linear structures, necessitating a new criterion. The 90% threshold was chosen as it is sufficient for functional electrical block since 100% completeness was not achieved in any cases.

### 2.5. Effectiveness and Procedural Outcomes

The primary effective outcome was long-term success rates of the procedure. Long-term ablation success was defined as freedom from atrial arrhythmia recurrence lasting longer than 30 s. The secondary effective outcome was acute ablation success, defined as achieving complete block of all 3 lines at the end of the index procedure. The safety outcome was cardiac perforation. Other measures included CF during ablation, fluoroscopic time and total procedure times.

### 2.6. Statistical Methods

The sample size for comparing recurrence rates was calculated based on the following assumptions: the Type I error was 0.05 (α), the statistical power was 0.8 (1-β) and the relative hazard ratio of the atrial tachyarrhythmia recurrence rate at 1 year was 0.5 (CF/Blind group). The hazard ratio was chosen based on clinical relevance. We hypothesized that effective transmural linear ablation could reduce the one-year recurrence rate from 30–60% to 15–30%. Data were analyzed according to the intention-to-treat principle. Categorical variables were presented as numbers and percentages and analyzed using a combination of the χ^2^ test and Fisher’s exact test. Continuous variables were presented as the mean and standard deviation and analyzed by Student’s *t*-test or the non-parametric Wilcoxon rank sum test. The cumulative risk of recurrent atrial tachyarrhythmia was estimated using Kaplan–Meier analysis and compared using the log-rank test. Multivariate Cox regression analysis was employed to assess the relationship between various ablation procedures and recurrence of atrial tachyarrhythmia. The proportional hazard assumption was evaluated by examining approximately parallel log-minus log survival curves during the follow-up period. The proportional hazard assumption was verified using Schoenfeld residuals. For variables where violations of the assumption were detected, we incorporated time-dependent covariates. All statistical tests were two-tailed, and a *p*-value of less than 0.05 was considered statistically significant. Statistical analyses were performed using SPSS (version 23, SPSS Institute, Inc., Chicago, IL, USA).

## 3. Results

### 3.1. Overall Ablation Index

**Figure 2:**
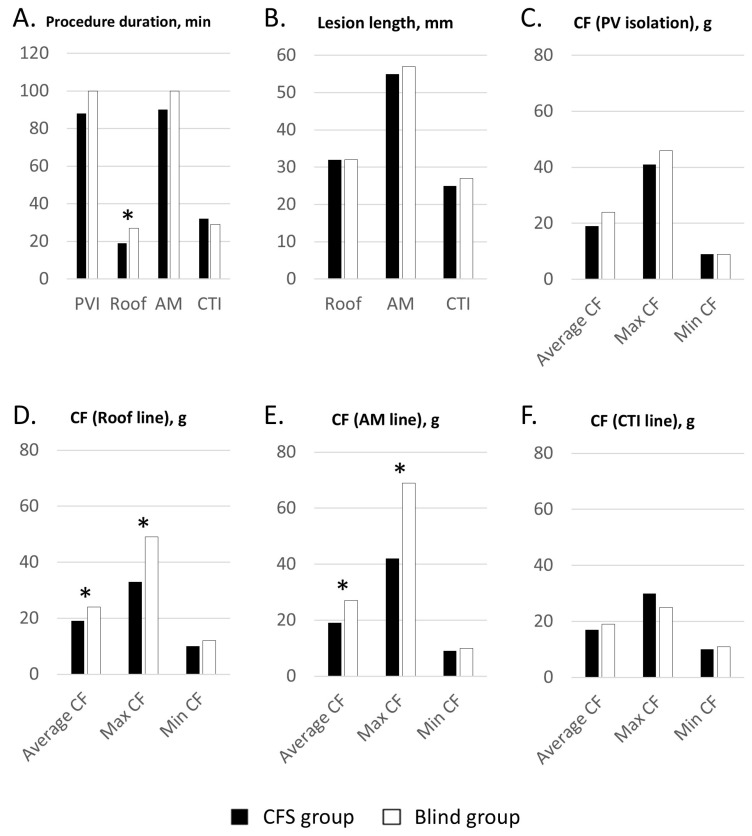
Procedural index and contact force information. * Significant value (*p* < 0.05). PVI = pulmonary vein isolation; AM = anteromitral; CTI = cavotricuspid isthmus; CF = contact force; Max = maximal; Min = minimal.

A total of 62 consecutive patients with drug-refractory PerAF were enrolled (58 ± 10 years old, 77% male) (Table 1). Acute successful block rates of the roof and AM line were 100% and 97% in the CFS group (n = 33) and 90% and 93% in the Blind group (n = 29), respectively. That of the CTI line was 100% in both groups. The time for linear ablation was reduced in the CFS group, especially the roof line (total linear ablation: 87 ± 52 vs. 104 ± 59, *p* = 0.046; roof line: 19 ± 14 vs. 27 ± 15 min, *p* = 0.031; AM line: 36 ± 22 vs. 48 ± 28 min, *p* = 0.068; CTI line 32 ± 16 vs. 29 ± 16, *p* = 0.478) (Figure 2). Fluoroscopic time during linear ablation was significantly shorter in the CFS group compared to the Blind group (8 ± 3 vs. 13 ± 6 min, *p* = 0.038).

### 3.2. Contact Force Index

The average CF and maximal CF at the roof line were significantly higher in the Blind group. On the AM line, the average CF and maximal CF were also significantly higher in the Blind group. The overall minimum CF was not different. The maximal CF for the safety endpoint was significantly lower in the CFS group (AM: 42 vs. 69 g, *p* < 0.001; roof: 33 vs. 49 g, *p* = 0.003) than in the Blind group.

### 3.3. LGE on CMR

**Figure 3:**
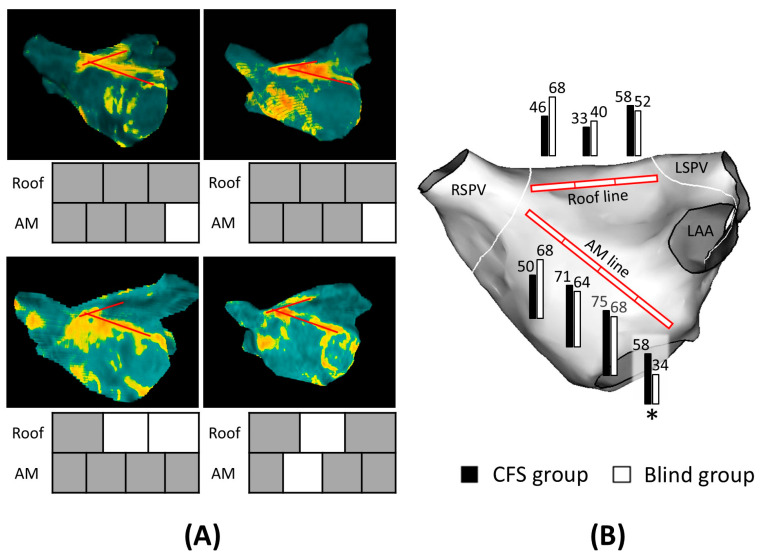
(**A**) Example of left atrial late gadolinium enhancement (LGE) analysis in cardiac magnetic resonance. The red line marks the roof and anteromitral ablation lines. LGE formation is considered complete if more than 80% of the line is filled with yellow- to orange-colored LGE. In this case, the completed areas are displayed in gray within the square below the figure. If LGE formation is less than 80%, the line segment is shown in white. AM = anteromitral. (**B**) The percentage of LGE completion for each line is provided to indicate the extent of scar formation along the roof and AM lines. Numbers are in %. * Significant value (*p* < 0.05). RSPV = right superior pulmonary vein; LSPV = left superior pulmonary vein; LAA = left atrial appendage; AM = anteromitral.

Full LA-LGE line achievement on CMR at one year did not differ significantly between the two groups. Specifically, the AM line was completed in 17% of cases in the CFS group versus 36% in the Blind group, and the roof line was completed in 29% versus 24%, respectively (*p* = NS). Figure 3A shows a post-MRI analysis example. In the figure, areas with sufficient LGE on the line are shaded gray while regions below the threshold baseline are highlighted as blank. We further compared the extent of LGE completion at each site (Figure 3B). While no significant difference in LGE formation was observed for the roof line, the CFS group demonstrated significantly better LGE formation on the left lateral side of the AM line.

### 3.4. Clinical Outcome

**Figure 4:**
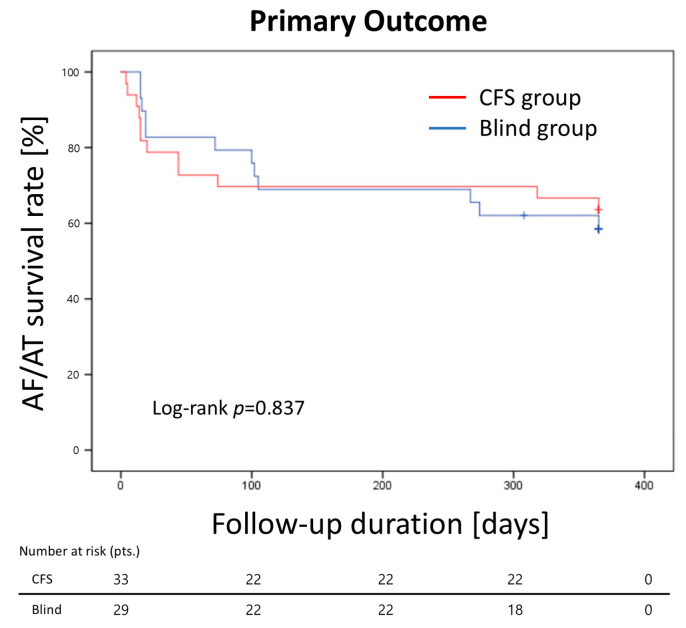
Atrial arrhythmia recurrence after catheter ablation as primary effective outcome. CFS = contact force sensing; AF = atrial fibrillation; AT = atrial tachycardia.

The Kaplan–Meier estimates of AF/AT-free survival after ablation procedures were 63.6% in the CFS group and 58.6% in the Blind group (log-rank *p* = 0.837) (Figure 4).

## 4. Discussion

The use of CF during linear ablation in patients with AF improved procedural safety by reducing the time required to achieve block and prevent excessive CF. However, the formation of permanent lesions as assessed by CMR with LGE did not differ between the two groups, and CF-guided linear ablation did not result in improved clinical outcomes.

### 4.1. Advantages of Contact Force in Linear Ablation

The benefits of using CF in PV isolation procedures are well established [10,11,12]. The pulmonary veins (PVs) present unique challenges due to their complex and unstable anatomy during catheter access [15,16]. CF technology has addressed many limitations associated with conventional procedures relying solely on 3D imaging. This benefit allows for more precise delivery of ablation energy to the correct location. Specifically, CF technology reduces the time required to achieve block during linear ablation, allowing for more precise and efficient energy delivery. Furthermore, CF monitoring enhances procedural safety by providing real-time feedback on catheter–tissue interaction, which helps avoid under- or over-ablation. CF monitoring plays a crucial role in enhancing procedural safety. In this study, excessive CF raised concerns about the potential for perforation, highlighting the importance of maintaining appropriate contact levels. CF monitoring is shown to improve safety during ablation [17,18]. Several large registry studies examining trends in radiofrequency catheter ablation have reported a reduction in perforation complications over time [19]. The use of CF technology has contributed significantly to this improvement.

### 4.2. Limitations of Contact Force in Linear Ablation

Despite its benefits, the utility of CF technology outside the PV region to improve clinical outcomes remains uncertain. In this study, success rates for achieving block and CMR-detected lesions did not differ significantly between the CF and Blind groups during linear ablation, suggesting that CF monitoring may be less critical for linear ablation compared to PV isolation. Maintaining optimal contact in areas such as the roof and left atrial anterior regions may be less challenging than in the PV region due to their relatively simpler anatomy.

### 4.3. LGE as a Marker for Ablation Effectiveness and the Role of Contact Force

LGE detected on CMR does not necessarily indicate a permanent electrical block. However, fibrosis is unlikely to develop without effective ablation. We used LGE as a marker for the effectiveness of CF monitoring and conducted site-specific analyses to evaluate its impact. The presence of residual gaps along the ablation line, where the scar failed to fully develop, was associated with recurrent arrhythmia [20]. Certain sites showed increased LGE in the CFS group, but the difference between groups was not significant. This lack of significance is attributed to insufficient CF being compensated by longer ablation times to achieve block. Additionally, LGE formation was reduced at the left end of the roof line and AM line in both groups. These findings suggest that increased focus on these regions may be necessary to ensure complete lesion formation. Transmural lesion formation is determined by multiple factors, including CF, duration of energy application per point, and power [21]. In this study, the ablation power was standardized at 30 W for all regions, and energy settings were not considered. Further investigation is warranted to elucidate the effects of advanced wattage settings on the linear ablation.

### 4.4. The Clinical Impact of Linear Ablation

This study aimed to evaluate the clinical effect of additional linear ablation for PerAF. In theory, linear ablation should prevent post-ablation left atrial macroreentry tachycardia and reduce arrhythmia burden [22,23,24,25]. However, clinical studies have not consistently demonstrated its effectiveness, but most of these studies were conducted before the adoption of CF technology [26,27]. Advocates for linear ablation highlight the technical challenges associated with achieving durable outcomes. Maintaining permanent linear block is difficult due to the long length of the ablation line and the presence of non-contact areas, which may result in gap-induced atrial tachycardia. In our study, we hypothesized that CF-sensing catheters, which have improved outcomes in PVI, could similarly enhance the effectiveness of linear ablation. However, we observed no significant differences in the creation of permanent lesions between the two groups nor any difference in clinical outcomes. Further research is needed to directly compare the efficacy of additional linear ablation with PVI without linear ablation using a CF-sensing catheter to validate the clinical value of linear ablation. Although no significant differences in clinical outcomes were identified between the two groups, these findings provide valuable insights into catheter ablation for patients with PerAF. This highlights the ongoing need to enhance our understanding of optimal ablation techniques in PerAF.

### 4.5. Study Limitation

The main limitation of this study is the small study population. Due to the high cost of CMR in Korea, a small number of patients were studied, and the study was conducted at a single center for CMR image quality and protocol uniformity. Due to this study design, the operators were not blinded to the randomization group, which may have affected the results of the study. All procedures were performed by experienced operators with previous exposure to CFS catheters, so the results may have been different if they were used by less experienced operators.

## 5. Conclusions

In additional linear ablation after PVI for patients with PerAF, the CF-sensing technique improved safety and reduced the procedure time to achieve block. However, it did not impact clinical outcomes or permanent full linear lesion formation in CMR.

## Figures and Tables

**Table 1:** Baseline characteristics of study population.

	Total n = 62	CFS Group n =33	Blind Group n = 29	*p*-Value
Age, years	58 ± 10	58 ± 9	58 ± 11	0.963
Male gender (%)	48 (77)	26 (79)	22 (76)	0.510
CHADS2 VASc score	1.6 ± 1.4	1.3 ± 1.1	1.9 ± 1.6	0.101
Body mass index, kg/m^2^	25 ± 3	25 ± 2	26 ± 3	0.598
Comorbidity				
Hypertension (%)	25 (40)	10 (30)	15 (52)	0.073
Diabetes mellitus (%)	6 (10)	2 (6)	4 (14)	0.275
Dyslipidemia (%)	9 (14)	4 (12)	5 (17)	0.114
Myocardial infarction (%)	0	0	0	
Cerebrovascular accident (%)	9 (15)	5 (15)	4 (14)	0.585
History of heart failure (%)	13 (21)	7 (21)	6 (21)	0.604
Chronic kidney disease (%)	0	0	0	
Echocardiographic data				
LV ejection fraction, %	52 ± 9	54 ± 9	51 ± 8	0.297
LA AP diameter, mm	43 ± 5	43 ± 4	43 ± 6	0.925
E/e’ ratio	9 ± 3	8 ± 3	9 ± 4	0.865

Data are presented as n (%) or mean ± standard deviation and were analyzed using χ^2^ test and unpaired two-tailed *t*-test or Fisher’s exact test. AF = atrial fibrillation; CMR = cardiac magnetic resonance; LGE = late gadolinium enhancement; LA = left atrial; LV = left ventricular; PASP = pulmonary artery systolic pressure; LAP = left atrial pressure.

## Data Availability

The datasets used and/or analysed during the current study available from the corresponding author on reasonable request.
